# Supplementary material for: End Sequence Analysis Toolkit (ESAT) expands the extractable information from single-cell RNA-seq data
Source: Genome Res. 2016 Oct;26(10):1397–410. doi: 10.1101/gr.207902.116 (PMC5052061; doi:10.1101/gr.207902.116)
Supplement: Supplemental Material [file supp_gr.207902.116_Supp_Materials.tar.gz › ESAT-esat_v0.1/doc/html/interfacebroad_1_1core_1_1annotation_1_1_lightweight_genomic_annotation.html]

ESAT: broad.core.annotation.LightweightGenomicAnnotation Interface Reference


ESAT


- Main Page
- Related Pages
- Packages
- Classes
- Files

- Class List
- Class Index
- Class Hierarchy
- Class Members

All Classes Namespaces Files Functions Variables Enumerator Pages

- broad
- core
- annotation
- LightweightGenomicAnnotation

Public Member Functions |
List of all members

broad.core.annotation.LightweightGenomicAnnotation Interface Reference

Inheritance diagram for broad.core.annotation.LightweightGenomicAnnotation:

[legend]

Collaboration diagram for broad.core.annotation.LightweightGenomicAnnotation:

[legend]

|  |  |
| --- | --- |
| Public Member Functions | |
| int | getStart () |
|  | |
| int | getEnd () |
|  | |
| long | getMiddle () |
|  | |
| double | getScore () |
|  | |
| String | getName () |
|  | |
| void | setScore (double score) |
|  | |
| void | setName (String name) |
|  | |
| Strand | getOrientation () |
|  | |
| void | setOrientation (String orientation) |
|  | |
| int | length () |
|  | |
| void | setStart (int start) |
|  | |
| void | setEnd (int end) |
|  | |
| String | getChromosome () |
|  | |
| void | setChromosome (String chr) |
|  | |
| boolean | inReversedOrientation () |
|  | |
| int | getDistanceTo (LightweightGenomicAnnotation other) |
|  | |
| String | toUCSC () |
|  | |
| int | getOverlap (LightweightGenomicAnnotation other) |
|  | |
| boolean | contains (LightweightGenomicAnnotation other) |
|  | |
| void | takeIntersection (LightweightGenomicAnnotation other) |
|  | |
| void | takeUnion (LightweightGenomicAnnotation other) |
|  | |
| void | stitchTo (LightweightGenomicAnnotation other) |
|  | |
| String | getChromosomeString () |
|  | |
| double | getExtraScore (int i) |
|  | |
| void | addExtraScore (double score) |
|  | |
| List< Double > | getExtraScores () |
|  | |
| void | removeExtraScores () |
|  | |
| boolean | almostEqual (LightweightGenomicAnnotation other, int fudge) |
|  | |
| Public Member Functions inherited from umms.core.annotation.Annotation | |
| int | getSAMStart () |
|  | |
| int | getSAMEnd () |
|  | |
| String | getReferenceName () |
|  | |
| String | getChr () |
|  | |
| Strand | getStrand () |
|  | |
| boolean | hasOrientation () |
|  | |
| boolean | isNegativeStrand () |
|  | |
| int | numBlocks () |
|  | |
| List<?extends Annotation > | getBlocks () |
|  | |
| List<?extends Annotation > | getBlocks (boolean oriented) |
|  | |
| int | getSize () |
|  | |
| int | size () |
|  | |
| int | getLengthOnReference () |
|  | |
| int | getOrientedStart () |
|  | |
| int | getOrientedEnd () |
|  | |
| boolean | isUnoriented () |
|  | |
| int | getReferenceCoordinateAtPosition (int positionInAnnotation) |
|  | |
| int | getPositionAtReferenceCoordinate (int referenceCoordinate) |
|  | |
| int | getReferenceCoordinateAtPosition (int positionInAnnotation, boolean ignoreOrientation) |
|  | |
| int | getPositionAtReferenceCoordinate (int referenceCoordinate, boolean ignoreOrientation) |
|  | |
| void | setOrientation (char orientation) |
|  | |
| void | setOrientation (Strand orientation) |
|  | |
| void | setOrientedStart (int orientedStart) |
|  | |
| void | setOrientedEnd (int orientedEnd) |
|  | |
| void | setReferenceName (String refName) |
|  | |
| boolean | equals (Annotation other) |
|  | |
| void | expand (int deltaStart, int deltaEnd) |
|  | |
| Annotation | trim (int deltaStart, int deltaEnd) |
|  | |
| void | shift (int delta) |
|  | |
| void | moveToCoordinate (int coordinateInReference) |
|  | |
| Annotation | copy () |
|  | |
| List< Annotation > | disect (Annotation a) |
|  | |
| List< Annotation > | disect (List<?extends Annotation > disectors) |
|  | |
| Annotation | minus (Annotation other) |
|  | |
| Annotation | minus (Collection<?extends Annotation > others) |
|  | |
| int | getDistanceTo (Annotation other) |
|  | |
| String | toBED () |
|  | |
| String | toBED (int r, int g, int b) |
|  | |
| String | toShortBED () |
|  | |
| String | toBEDGraph () |
|  | |
| String | getFullInfoString () |
|  | |
| boolean | overlaps (Annotation other, int buffer) |
|  | |
| boolean | overlaps (Collection<?extends Annotation > others, int buffer) |
|  | |
| boolean | overlaps (Annotation other) |
|  | |
| boolean | overlaps (Collection<?extends Annotation > others) |
|  | |
| boolean | overlaps (Annotation other, int buffer, boolean considerOrientation) |
|  | |
| boolean | overlaps (Annotation other, boolean considerOrientation) |
|  | |
| boolean | overlapsStranded (Annotation other) |
|  | |
| int | getOverlap (Annotation other) |
|  | |
| boolean | contains (Annotation other) |
|  | |
| Annotation | union (Annotation other) |
|  | |
| Annotation | intersect (Annotation other) |
|  | |
| List< Annotation > | intersect (List<?extends Annotation > annotations) |
|  | |
| int | compareToAnnotation (Annotation b) |
|  | |
| void | stitchTo (Annotation next) |
|  | |
| String | toCigar () |
|  | |
| boolean | fullyContains (Annotation annotation) |
|  | |
| Annotation | complement () |
|  | |
| boolean | equals (Annotation other, boolean useOrientation) |
|  | |
| Collection<?extends Annotation > | getSpliceConnections () |
|  | |
| int | getMidpoint () |
|  | |

## Member Function Documentation

|  |  |  |  |  |  |
| --- | --- | --- | --- | --- | --- |
| void broad.core.annotation.LightweightGenomicAnnotation.addExtraScore | ( | double | *score* | ) |  |

Adds a new extra score

Parameters
:   |  |  |
    | --- | --- |
    | score |  |

Implemented in broad.core.annotation.BasicLightweightAnnotation.

|  |  |  |  |
| --- | --- | --- | --- |
| boolean broad.core.annotation.LightweightGenomicAnnotation.almostEqual | ( | LightweightGenomicAnnotation | *other*, |
|  |  | int | *fudge* |
|  | ) |  |  |

Checks whether two annotations differ by a small (fudge) factor

Parameters
:   |  |  |
    | --- | --- |
    | other | The other annotation |
    | fudge | Maximum difference at either end or start to consider similar |

Returns

Implemented in broad.core.annotation.BasicLightweightAnnotation.

|  |  |  |  |  |  |
| --- | --- | --- | --- | --- | --- |
| boolean broad.core.annotation.LightweightGenomicAnnotation.contains | ( | LightweightGenomicAnnotation | *other* | ) |  |

Implemented in broad.core.annotation.BasicLightweightAnnotation.

|  |  |  |  |  |
| --- | --- | --- | --- | --- |
| String broad.core.annotation.LightweightGenomicAnnotation.getChromosome | ( |  | ) |  |

Implemented in broad.core.annotation.BasicLightweightAnnotation.

Here is the caller graph for this function:

|  |  |  |  |  |
| --- | --- | --- | --- | --- |
| String broad.core.annotation.LightweightGenomicAnnotation.getChromosomeString | ( |  | ) |  |

Returns the usual way to write a chromosome: chrSymbol if a chromosome or the full name of the scaffold

Implemented in broad.core.annotation.BasicLightweightAnnotation.

|  |  |  |  |  |  |
| --- | --- | --- | --- | --- | --- |
| int broad.core.annotation.LightweightGenomicAnnotation.getDistanceTo | ( | LightweightGenomicAnnotation | *other* | ) |  |

Calculates the distance to the another genomic annotation.

Returns
:   0 if the annotations overlap or the minimum between the edges otherwise.

Implemented in broad.core.annotation.BasicLightweightAnnotation.

Here is the caller graph for this function:

|  |  |  |  |  |
| --- | --- | --- | --- | --- |
| int broad.core.annotation.LightweightGenomicAnnotation.getEnd | ( |  | ) |  |

Returns
:   end of the annotation (exclusive)

Implements umms.core.annotation.Annotation.

|  |  |  |  |  |  |
| --- | --- | --- | --- | --- | --- |
| double broad.core.annotation.LightweightGenomicAnnotation.getExtraScore | ( | int | *i* | ) |  |

Returns the ith extra score

Implemented in broad.core.annotation.BasicLightweightAnnotation.

|  |  |  |  |  |
| --- | --- | --- | --- | --- |
| List<Double> broad.core.annotation.LightweightGenomicAnnotation.getExtraScores | ( |  | ) |  |

Returns
:   all extra scores

Implemented in broad.core.annotation.BasicLightweightAnnotation.

Here is the caller graph for this function:

|  |  |  |  |  |
| --- | --- | --- | --- | --- |
| long broad.core.annotation.LightweightGenomicAnnotation.getMiddle | ( |  | ) |  |

Implemented in broad.core.annotation.BasicLightweightAnnotation.

|  |  |  |  |  |
| --- | --- | --- | --- | --- |
| String broad.core.annotation.LightweightGenomicAnnotation.getName | ( |  | ) |  |

Returns
:   the name of the annotation

Implements umms.core.annotation.Annotation.

Here is the caller graph for this function:

|  |  |  |  |  |
| --- | --- | --- | --- | --- |
| Strand broad.core.annotation.LightweightGenomicAnnotation.getOrientation | ( |  | ) |  |

Returns
:   orientation or strand

Implements umms.core.annotation.Annotation.

Implemented in broad.core.annotation.GenomicAnnotation.

Here is the caller graph for this function:

|  |  |  |  |  |  |
| --- | --- | --- | --- | --- | --- |
| int broad.core.annotation.LightweightGenomicAnnotation.getOverlap | ( | LightweightGenomicAnnotation | *other* | ) |  |

Parameters
:   |  |  |
    | --- | --- |
    | other | GenomicAnnotation to check of overlap |

Returns
:   true if the current instance overlaps with the other one. Returns the number of bases that overlap between the two annotations.

Parameters
:   |  |  |
    | --- | --- |
    | other |  |

Returns

Implemented in broad.core.annotation.BasicLightweightAnnotation.

|  |  |  |  |  |
| --- | --- | --- | --- | --- |
| double broad.core.annotation.LightweightGenomicAnnotation.getScore | ( |  | ) |  |

Implements umms.core.annotation.Annotation.

Implemented in broad.core.annotation.BasicLightweightAnnotation, and broad.pda.datastructures.Alignments.

|  |  |  |  |  |
| --- | --- | --- | --- | --- |
| int broad.core.annotation.LightweightGenomicAnnotation.getStart | ( |  | ) |  |

Returns
:   start of the annotation (inclusive)

Implements umms.core.annotation.Annotation.

|  |  |  |  |  |
| --- | --- | --- | --- | --- |
| boolean broad.core.annotation.LightweightGenomicAnnotation.inReversedOrientation | ( |  | ) |  |

Implemented in broad.core.annotation.BasicLightweightAnnotation.

Here is the caller graph for this function:

|  |  |  |  |  |
| --- | --- | --- | --- | --- |
| int broad.core.annotation.LightweightGenomicAnnotation.length | ( |  | ) |  |

Returns
:   length of the Annotation; if it has blocks, the gaps between blocks are not included

Implements umms.core.annotation.Annotation.

Implemented in broad.core.annotation.BasicLightweightAnnotation, and broad.pda.datastructures.Alignments.

Here is the caller graph for this function:

|  |  |  |  |  |
| --- | --- | --- | --- | --- |
| void broad.core.annotation.LightweightGenomicAnnotation.removeExtraScores | ( |  | ) |  |

removes extra scores

Implemented in broad.core.annotation.BasicLightweightAnnotation.

|  |  |  |  |  |  |
| --- | --- | --- | --- | --- | --- |
| void broad.core.annotation.LightweightGenomicAnnotation.setChromosome | ( | String | *chr* | ) |  |

Implemented in broad.core.annotation.BasicLightweightAnnotation.

|  |  |  |  |  |  |
| --- | --- | --- | --- | --- | --- |
| void broad.core.annotation.LightweightGenomicAnnotation.setEnd | ( | int | *end* | ) |  |

Sets a new end position. Will delete and/or truncate blocks if the new end position is less than the old one. Will extend the last block if the new end position is greater than old one.

Parameters
:   |  |  |
    | --- | --- |
    | new | end position (ignoring orientation) of the annotation |

Implements umms.core.annotation.Annotation.

Here is the caller graph for this function:

|  |  |  |  |  |  |
| --- | --- | --- | --- | --- | --- |
| void broad.core.annotation.LightweightGenomicAnnotation.setName | ( | String | *name* | ) |  |

Parameters
:   |  |  |
    | --- | --- |
    | name | name of the annotation |

Implements umms.core.annotation.Annotation.

Here is the caller graph for this function:

|  |  |  |  |  |  |
| --- | --- | --- | --- | --- | --- |
| void broad.core.annotation.LightweightGenomicAnnotation.setOrientation | ( | String | *orientation* | ) |  |

Implemented in broad.core.annotation.BasicLightweightAnnotation, and broad.core.annotation.GenomicAnnotation.

|  |  |  |  |  |  |
| --- | --- | --- | --- | --- | --- |
| void broad.core.annotation.LightweightGenomicAnnotation.setScore | ( | double | *score* | ) |  |

Implements umms.core.annotation.Annotation.

Implemented in broad.core.annotation.BasicLightweightAnnotation.

|  |  |  |  |  |  |
| --- | --- | --- | --- | --- | --- |
| void broad.core.annotation.LightweightGenomicAnnotation.setStart | ( | int | *start* | ) |  |

Sets a new start position. Will delete and/or truncate blocks if the new start position is greater than the old one. Will extend the first block if the new start position is less than old one.

Parameters
:   |  |  |
    | --- | --- |
    | new | start position (ignoring orientation) of the annotation |

Implements umms.core.annotation.Annotation.

|  |  |  |  |  |  |
| --- | --- | --- | --- | --- | --- |
| void broad.core.annotation.LightweightGenomicAnnotation.stitchTo | ( | LightweightGenomicAnnotation | *other* | ) |  |

Enlarges this annotation by

Parameters
:   |  |  |
    | --- | --- |
    | other |  |

Implemented in broad.core.annotation.BasicLightweightAnnotation.

|  |  |  |  |  |  |
| --- | --- | --- | --- | --- | --- |
| void broad.core.annotation.LightweightGenomicAnnotation.takeIntersection | ( | LightweightGenomicAnnotation | *other* | ) |  |

Change the current instance to represent its intersection with the provided annotation

Parameters
:   |  |  |
    | --- | --- |
    | other |  |

Implemented in broad.core.annotation.BasicLightweightAnnotation.

|  |  |  |  |  |  |
| --- | --- | --- | --- | --- | --- |
| void broad.core.annotation.LightweightGenomicAnnotation.takeUnion | ( | LightweightGenomicAnnotation | *other* | ) |  |

Change the current instance to represent its union with the provided annotation

Parameters
:   |  |  |
    | --- | --- |
    | GenomicAnnotation | other |

Implemented in broad.core.annotation.BasicLightweightAnnotation.

|  |  |  |  |  |
| --- | --- | --- | --- | --- |
| String broad.core.annotation.LightweightGenomicAnnotation.toUCSC | ( |  | ) |  |

Similar to getLocationString()

See also
:   getLocationString and provided only for backwards compatibility.

Implements umms.core.annotation.Annotation.

Implemented in broad.core.annotation.BasicLightweightAnnotation, and broad.pda.datastructures.Alignments.

---

The documentation for this interface was generated from the following file:

- java/broad/core/annotation/LightweightGenomicAnnotation.java


---

Generated on Fri Aug 15 2014 15:10:51 for ESAT by  

 1.8.7
